# The genome of *Chenopodium ficifolium*: developing genetic resources and a diploid model system for allotetraploid quinoa

**DOI:** 10.1093/g3journal/jkaf162

**Published:** 2025-07-18

**Authors:** Clayton D Ludwig, Peter J Maughan, Eric N Jellen, Thomas M Davis

**Affiliations:** Department of Agriculture, Nutrition, and Food Systems, University of New Hampshire, Durham, NH 03824, United States; Department of Plant and Wildlife Sciences, College of Life Sciences, Brigham Young University, Provo, UT 84602, United States; Department of Plant and Wildlife Sciences, College of Life Sciences, Brigham Young University, Provo, UT 84602, United States; Department of Agriculture, Nutrition, and Food Systems, University of New Hampshire, Durham, NH 03824, United States

**Keywords:** genome, assembly, annotation, *Chenopodium ficifolium*, quinoa, FTL, GWAS

## Abstract

High-quality nuclear, chloroplast, and preliminary mitochondrial genomes have been assembled and annotated for the B genome diploid (BB: 2*n* = 2*x* = 18) figleaf goosefoot (*Chenopodium ficifolium*). The primary objective was to advance a simplified model system for genetic characterization and improvement of allotetraploid (AABB: 2*n* = 4*x* = 36) quinoa (*Chenopodium quinoa*), a nutritionally valuable, halophytic orphan crop. In addition to its diploidy and favorably small genome size, the *C. ficifolium* model provides a shorter generational period and smaller overall plant size as compared to *C. quinoa*, while displaying relevant agronomic trait variations amenable to gene–trait association studies. The *C. ficifolium* “Portsmouth” nuclear genome was sequenced using PacBio HiFi long-read technology and assembled using Hifiasm. After manual adjustments, the final ChenoFicP_1.0 assembly consisted of 9 pseudochromosomes spanning 730 Mbp, while 22,617 genes were identified and annotated. BUSCO analyses indicated a nuclear genome completeness of 97.5% and a proteome and transcriptome completeness of 98.4%. The chloroplast genome assembly detected 2 equally represented structural haplotypes differing in the orientation of the short single copy region relative to the long single copy region. Phylogenetic and parentage analyses pointed to an unspecified AA diploid species and away from *C. ficifolium* as the likely maternal chloroplast and mitochondrial genome donor(s) during the initial tetraploidization event in the *C. quinoa* lineage. Using the new ChenoFicP_1.0 reference genome, a genome-wide association study was performed on a previously studied *C. ficifolium* F2 population to further define region(s) implicated in the control of 3 key agronomic traits: days to flowering, plant height, and branch number. This analysis localized control of all 3 traits to a 7-Mb interval on pseudochromosome Cf4. This region contains ∼770 genes, including the *FTL1* locus, thus confirming and extending our prior, single-marker analysis showing association of these 3 traits with an *FTL1* amplicon length polymorphism. The use of these data to further develop *C. ficifolium* as a model species for genetics and breeding of quinoa serves to expand knowledge and germplasm resources for quinoa improvement.

## Introduction

*Chenopodium ficifolium* (figleaf goosefoot) is a weedy, Old-World B genome diploid (BB: 2*n* = 2*x* = 18) relative of the cultivated allotetraploid (AABB: 2*n* = 4*x* = 36) pseudocereal crop quinoa (*Chenopodium quinoa* Willd.) (Walsh et al. 2015; Kolano et al. 2016) and its wild sister AABB tetraploid *Chenopodium berlandieri* (Walsh et al. 2015). The genus *Chenopodium* now resides in the family Amaranthaceae, its former Chenopodiaceae family home having recently been subsumed into the Amaranthaceae (The Angiosperm Phylogeny Group et al. 2016), which also hosts the relevant comparator *Beta vulgaris* (sugar beet). Putatively originating from Eurasia (Walsh et al. 2015), figleaf goosefoot is now widespread in North America, both as a weed of human disturbance and as a naturalized part of disturbed ecosystems. It is 1 of 2 recognized potential contributors, or a close relative thereof, of the B subgenome to the *C. quinoa* lineage during its initial tetraploidization event, the other B subgenome candidate being diploid *Chenopodium suecicum* (Jellen et al. 2011; Mandák et al. 2012; Walsh et al. 2015; Jarvis et al. 2017). Because figleaf goosefoot potentially contributed the B subgenome to quinoa, or is an extant sister species of the contributor, it is a highly relevant candidate to explore as a diploid model species in relation to quinoa's genetic characterization and breeding, and its study may shed light on specific genes governing domestication-related traits in quinoa (Jellen et al. 2015; Walsh et al. 2015; Kolano et al. 2016; Subedi et al. 2021).

In the absence of a history of intensive breeding, *C. quinoa* has been categorized as an “orphan crop” (Lemmon et al. 2018; Kumar and Bhalothia 2020). However, quinoa is receiving increasing interest from consumers due to its high nutritional value and from agronomists and breeders due to its drought and salt tolerance (Gorinstein et al. 2002; Rojas et al. 2009; Vega-Gálvez et al. 2010; Jacobsen 2011). These and other attributes make quinoa an attractive candidate for production in regions that cannot sustain the major grain crops. Furthermore, quinoa has the potential to serve as a valuable secondary crop, thereby contributing to food security. Thus, it is an attractive candidate for increased production, justifying investment in development of novel genetics and methods to streamline the breeding process (Jacobsen 2003; Bazile et al. 2016).

Using *C. ficifolium* as a diploid model species has the potential to accelerate trait dissection and gene identification due to its comparative genetic simplicity; shorter generation time of ∼40 d compared to quinoa's 90 to 120 d (Jacobsen 1997); profuse flower, pollen, and seed production; and smaller plant size facilitating growth chamber cultivation (Subedi et al. 2021). Building upon prior, foundational studies (Štorchová et al. 2015, 2019), the targeted development of *C. ficifolium* as a diploid model species was initiated (Subedi 2020; Subedi et al. 2021) with a focus on (i) producing intraspecific hybrids and segregating progenies; (ii) investigating segregation in domestication-related traits of relevance to *C. quinoa* breeding programs; and (iii) detecting gene–trait associations for key genes and traits.

In an F2 segregating population from an initial cross involving *C. ficifolium* accessions from Portsmouth (“P”), New Hampshire, United States, and Quebec City (“QC”), Quebec, Canada, allelic variation in the *Flowering Locus T-Like 1* (*FTL1*) marker locus was found to be associated with variation in days to flower, plant height, and branch number (Subedi 2020; Subedi et al. 2021). Using previously designed primers (Cháb et al. 2008; Štorchová et al. 2015) to target and conveniently genotype an indel polymorphism within the *FTL1* gene, it was found that F2 plants homozygous for the “P” allele flowered on average 12 d earlier than plants homozygous for the “QC” allele and 2 d earlier than heterozygotes (Subedi 2020; Subedi et al. 2021). As a likely consequence of their decreased maturation time, “P” allele homozygotes were also significantly shorter and had fewer branches than plants of the alternate genotypes. These results strongly implicated the *FTL1* chromosomal region in the control of 3 agronomically important traits, but did not rule out the possibility of influence from other genomic regions. While genotype–phenotype relationships involving *FTL1* must be further studied at both diploid and the tetraploid levels, this research demonstrates the potential value of *C. ficifolium* as a model system in which to accelerate discovery and characterization of gene–trait relationships. The goal of the work reported here is to expand knowledge and establish foundational genomic resources for the *C. ficifolium* model system.

## Materials and methods

### Germplasm

All plant cultivation and phenotyping activities were conducted at the New Hampshire Agricultural Experiment Station at the University of New Hampshire (UNH). Two figleaf goosefoot accessions were employed in this study, both of which have been deposited as seed samples with the USDA North Central Plant Introduction Station at Ames, Iowa. The “P” accession (PI 698433) was collected by Erin Neff and Thomas Davis in Portsmouth, New Hampshire, United States (Neff 2017), while the “QC” accession (PI 698434) was collected by Thomas Davis from Quebec City, Quebec, Canada. Crosses between the “P” and “QC” accessions to generate F1 and F2 populations, and confirmation of parentage using informative *FTL1* amplicons, were as previously described (Subedi et al. 2021). For purposes of comparison, we also employed in-house-numbered accessions 302-A of AA diploid *Chenopodium foggii* and RB6 of AABB allotetraploid *C. berlandieri* var. *macrocalycium*, which had been collected by Erin Neff and Thomas Davis in southern Maine (Neff 2017; Neff et al. 2018) and in Rye Beach, New Hampshire, respectively, and *C. quinoa* accession QQ065 (PI 614880), obtained from USDA NPGS.

### DNA isolation and genome sequencing

The figleaf goosefoot “P” accession was used to construct the primary de novo reference genome assembly. Using leaf tissues provided by the UNH investigators, DNA from the “P” accession was isolated at Brigham Young University (BYU) using methods designed to yield high molecular weight (HMW) DNA from plant tissues (Vaillancourt and Buell 2019). The “P” accession HMW DNA was sequenced at the BYU DNA Sequencing Center (DNASC) using the PacBio HiFi platform (Eid et al. 2009). In addition, this DNA was used at UNH to generate Nanopore sequence on an Oxford Nanopore Technologies (ONT) R9.4.1 flow cell using a library prepared with a rapid sequencing kit (SQK-RAD004) following methods described by Nanopore entitled “Rapid sequencing gDNA—whole genome amplification.”

Illumina sequence was generated at the UNH Hubbard Center for Genome Studies (HCGS) for the following: for the “P” and “QC” accessions of *C. ficifolium*; for 35 F2 generation individuals descended from a “P” × “QC” cross that was previously studied and described (Subedi et al. 2021); for the RB6 accession of *C. berlandieri* var. *macrocalycium*; and for the 302-A accession of *C. foggii*. DNA for Illumina sequencing was isolated at UNH (Subedi et al. 2021) using a CTAB method described by Torres et al. (1993).

### Nuclear genome assembly

PacBio reads were assembled at BYU using Hifiasm version v0.18.5-r499 (Cheng et al. 2021, 2022) with default settings. Subsequent correction, annotation, and analyses were conducted at UNH. Inspector (Chen et al. 2021) was used to detect and correct errors in the Hifiasm assembly followed by manual examination and adjustments, yielding the *C. ficifolium* version 1.0 nuclear reference genome designated as ChenoFicP_1.0. Finally, BUSCO (Manni et al. 2021) and dependencies (Stanke et al. 2008; Camacho et al. 2009; Eddy 2011; Mirarab et al. 2012; Levy Karin et al. 2020; Li, 2023) using default settings and the embryophyta_odb10 database were used to calculate nuclear genome completeness. Telomeres were identified within pseudochromosomes (PCs) with the quarTeT toolkit program TeloExplorer (Lin et al. 2023) and within unplaced contigs using tidk (Brown et al., 2023). Centromeric regions were identified using the tool CentroMiner which is supplied with the quarTeT toolkit (Lin et al. 2023). Nuclear genome assembly data were visualized using Circos (Krzywinski et al. 2009) in combination with the deepStats toolkit (Richard 2019) and BEDOPS (Neph et al. 2012) for track generation.

### Nuclear genome annotation

Once ChenoFicP_1.0 was finalized, repetitive elements were identified using RepeatModeler (Flynn et al. 2020). Those repetitive elements were then masked using RepeatMasker (Tarailo-graovac and Chen 2009; Flynn et al. 2020; Storer et al. 2021) as the first step in an annotation pipeline patterned after Card et al. (2019). The RepeatMasker scripts calcDivergenceFromAlign.pl and createRepeatLandscape.pl were used to analyze output files, determine repetitive element content, and produce data visualizations. A BRAKER IsoSeq compatible singularity container (Brůna et al. 2024; Brůna et al. 2021; Gabriel et al. 2021; Stanke et.al. 2008; Stanke et. al. 2006), was passed to the fasta file containing soft-masked repetitive elements and Pacific Biosciences IsoSeq data aligned to the genome using minimap2 (Li 2018). BRAKER flags were set to use the Viridiplantae OrthoDB database (Kuznetsov et al. 2023) for protein sequence and the Embryophyta odb10 database (Manni et al. 2021) as the BUSCO lineage.

The resulting GFF3 annotation file was passed to MAKER (Holt and Yandell 2011) as a prediction GFF file, as were the cds-transcripts and proteins files which were used for expressed sequence tag evidence and protein homology evidence, respectively. MAKER was used to update annotations and to provide annotation edit distance (AED) scores. AED values for the final annotation file were produced using AED_cdf_generator.pl (https://github.com/mscampbell). Once complete, the output files for each PC were merged to create 1 contiguous file. This output file contained gene predictions, however without functional annotations. To generate functional annotatio and GO terms, BLASTP (Johnson et al. 2008) utilizing the UniProtKB/Swiss-Prot database (UniProt Consortium 2015), and InterProScan (Jones et al. 2014) were supplied the protein sequences generated by MAKER. The outputs from these tools were then combined using the maker_functional_gff and ipr_update_gff scripts. Finally, the resulting GFF3 file was sorted and standardized using AGAT (Dainat et al. 2020), producing the final GFF3 annotation file.

A genome-wide synteny analysis was performed comparing the ChenoFicP_1.0 assembly to the B subgenome of the *C. quinoa* V2 reference genome (Rey et al. 2023) and to the *B. vulgaris* EL10.1 reference genome (McGrath et al. 2023). As a comember of the Amaranthaceae family, diploid (2*n* = 2*x* = 18) *B. vulgaris* (sugar beet) has been used in previous genomic comparisons with quinoa (Jarvis et al. 2017; Rey et al. 2023). To produce the synteny analysis, *C. quinoa* annotations and protein fasta files were downloaded from CoGe (id60716) and *B. vulgaris* files from NCBI (GCF_002917755.1). Annotation files were converted to BED format via agat_convert_sp_gff2bed.pl (Dainat et al. 2020), before being manually rearranged to comply with requirements for MCScanX_h (Wang et al. 2012). BlastP was used to compare the proteome of *C. ficifolium* with those of *B. vulgaris* and *C. quinoa* (Camacho et al. 2009), yielding 2 blast output files which were merged and passed to MCScanX_h, along with BED files containing positional information for the genes in the blast files. For this analysis, MCScanX used default filtering parameters. SynVisio (Bandi and Gutwin 2020) was used to visualize the resulting files, illuminating rearrangements and structural variations.

### Chloroplast genome assembly and annotation

The “P” chloroplast genome was initially assembled from extracted PacBio reads using GetOrganelle v1.7.6.1 (Jin et al. 2020), with dependencies SPAdes (Bankevich et al. 2012), Bowtie2 (Langmead and Salzberg 2012), BLAST+ (Camacho et al. 2009), and Bandage (Wick et al. 2015), and annotated with the online tool GeSeq (Tillich et al. 2017). GetOrganelle was called using get_organelle_from_reads.py and was supplied with raw PacBio reads using the unpaired “-u” flag. Embplant_pt was selected as the genome type, and the target genome size was set to 150 kb which is the approximate size of the *C. quinoa* chloroplast genome (Hong et al. 2017). For the purpose of determining the relative abundance of each detected haplotype of the “P” chloroplast genome based on the numbers of supporting Nanopore reads, Cp-hap (Wang et al. 2019) passed reads produced via an ONT R9.4.1 flow cell as previously noted. The final output file produced from GetOrganelle was passed to GeSeq (https://chlorobox.mpimp-golm.mpg.de/geseq.html) (Tillich et al. 2017) for visualization and annotation.

### Mitochondrial genome assembly and annotation

A contig from the Hifiasm assembly was identified as mitochondrial by aligning all unplaced contigs to the quinoa mitochondrial reference sequence NC_041093.1 (Maughan et al. 2019) with BWA-MEM (Li and Durbin 2009) and manually selecting a long, well-aligned contig. This contig was used as a seed sequence and passed to a de novo organelle assembly pipeline described by Jarvis et al. (2022) to assemble the mitochondrial genome. The pipeline was used in 2 iterations, initially using the HiFi dataset which produced a contig that was passed to the mitochondrial assembly pipeline in conjunction with the long-read Nanopore dataset. The Nanopore dataset was then aligned to this contig using minimap2 (Li, 2018), reads matching GC content and length thresholds were stripped from the alignment file via SeqKit (Shen et al. 2016), and these reads were passed to Canu (Koren et al. 2017) for de novo assembly. Nanopore reads were later used to resolve assembly structure via comparing alignments to the draft mitogenome assembly. The output contig from Canu was manually circularized by locating regions at the start and end of the file that shared 100% sequence identity. The circularized version of the assembly was polished using NextPolish (Hu et al. 2020) and the PacBio read set to correct single nucleotide errors. Annotation was performed by GeSeq. Importantly, GeSeq was set to pass third-party mitochondrial NCBI RefSeqs from both *C. quinoa* (NC_041093.1) and *B. vulgaris* subsp. *vulgaris* (NC_002511.2) to BLAT (Kent 2002) which used annotations from those genomes to locate and annotate homologs within the *C. ficifolium* mitogenome.

### Chloroplast and mitochondrial inheritance patterns in *C. ficifolium*

Chloroplast and mitochondrial reference-guided assemblies for “QC,” 1 F1 individual, and 4 F2 individuals were constructed and aligned to the respective “P” organelle genomes using BWA-MEM (Li and Durbin 2009) to identify heritable organelle polymorphisms differentiating the 2 parents. The resulting SAM files were then converted to BAM, sorted, and indexed, and a consensus was called using the respective samtools modules (Danecek et al. 2021). Once BAM files for each individual were converted to fasta, freebayes-parallel (Garrison and Marth 2012; Tange 2018) was used to call variants. Freebayes-parallel was invoked with options to produce a gVCF file, include genotype qualities, use best n alleles set to 4, and cap read depth to 5,000. VCF files were filtered to remove low-quality calls using vcffilter from vcflib (Garrison et al. 2022) and bcftools (Danecek et al. 2021). Sites or calls were pruned from the chloroplast multisample vcf file if (i) the average depth across all samples was <100; (ii) or the site quality was <998; (iii) or the genotype quality phred score was <30; (iv) or depth for a given sample was below 50; (v) or more than half of individuals were not represented; (vi) or the allele balance was <25% or >75%; (vii) or there was a discrepancy between the number of paired and unpaired reads aligned. The vcf file generated for the mitochondrial genome using the same samples was filtered using the same criteria with the addition of a cap of 3,000 on the average depth per sample. The filtering criteria and script were based on those provided by http://www.ddocent.com/filtering/. The resulting filtered vcf files were visualized with IGV (Thorvaldsdóttir et al. 2013).

### Chloroplast and mitochondrial genome ancestries

To illuminate the possible role of *C. ficifolium* in the cpDNA ancestries of allotetraploids *C. quinoa* and *C. berlandieri*, a minimal phylogenetic tree was generated using 6 chloroplast genome (cpDNA) assemblies representing 4 *Chenopodium* species and including beet (*B. vulgaris*) as an outgroup. The *C. quinoa* and *B. vulgaris* chloroplast genomes used were NCBI accessions MK159176.1 and OU343016.1, respectively. All other accessions included in this analysis were assembled by us de novo from raw reads using GetOrganelle (Jin et al. 2020). We assembled the *C. berlandieri* chloroplast genome from Illumina 2 × 150 PE reads previously uploaded to NCBI by Mark Samuels from the University of Montreal (accession: PRJNA895488) (Samuels et al. 2023). The *C. ficifolium* “P” chloroplast genome was de novo assembled using sequence data extracted from the PacBio dataset as described above, while the *C. ficifolium* “QC” and the *C. foggii* 302-A chloroplast genomes were assembled from Illumina data produced at the UNH-HCGS. These assemblies were aligned using Mafft (Katoh and Standley 2013) with default (auto) settings, and a tree was produced using RAxML V. 8.2.12 with the GTRCAT model (Stamatakis 2014). Trees were visualized with TreeViewer (Bianchini and Sánchez-Baracaldo 2023).

For a parallel purpose, a minimal mitochondrial phylogeny was produced by aligning raw reads, either generated by the HCGS for *C. foggii* 302-A and “P” and “QC” *C. ficifolium* accessions or downloaded from NCBI, to the quinoa mitochondrial reference genome NC_041093.1 (Maughan et al. 2019). *B. vulgaris* (SRR6305245) (McGrath et al. 2023) and *C. berlandieri* (PRJNA895488) (Samuels et al. 2023) read sets were downloaded from NCBI. All read data used in this analysis were trimmed and cleaned with Trimmomatic (Bolger et al. 2014) using the primer sequence file “TruSeq3-PE-2.fa” invoked with the following settings: ILLUMINACLIP:TruSeq3-PE-2.fa:2:30:10 LEADING:3 TRAILING:3 SLIDINGWINDOW:4:15 MINLEN:36. FastQC (https://www.bioinformatics.babraham.ac.uk/projects/fastqc/) was used to visualize and validate the Trimmomatic output. These reads were then aligned to the quinoa mitochondrial reference genome (Maughan et al. 2019) using BWA-MEM (Li and Durbin 2009). Using samtools view, sort, index, and consensus (Danecek et al. 2021), the BWA-produced SAM file was converted to a fasta file containing the consensus sequence. These 6 fasta files, those produced from reads plus the quinoa mitogenome file used as a reference in the alignment process, were merged into a multi-fasta, and Mafft (Katoh and Standley 2013) was used with default (auto) settings to produce an MSA. A tree was produced using RAxML V. 8.2.12 (Stamatakis 2014) with the GTRCAT model and *B. vulgaris* specified as the outgroup.

### Genome-wide association studies

A subset of 21 “P” × “QC” F2 individuals chosen from the 35 sequenced at the HCGS on the basis of high read depth was used in a genome-wide association study (GWAS) analysis to locate regions throughout the genome implicated in the control of branch angle, internode length, flowering time, plant height, and number of lateral branches. BWA-MEM was used to align reads from F2 individuals to the *C. ficifolium* “P” V1.0 reference genome. The resulting sam files were converted to bam, sorted, and indexed via samtools (Danecek et al. 2021) and read depths visualized with WGSCoveragePlotter (Lindenbaum 2015). GATK AddOrReplaceReadGroups (Van der Auwera and O’Connor 2020) was called to change read group IDs to reflect the sample name to make variant calling possible. Freebayes-parallel (Garrison and Marth 2012; Tange 2018) was used to generate a vcf file containing both indel and SNP variants for all 35 initial individuals. The resulting vcf file was filtered to remove low-quality calls following guidelines from http://www.ddocent.com/filtering/. The filtered vcf data in conjunction with phenotypic data collected by Subedi (2020) (Supplementary Table 1) and name and positional data extracted from the GFF3 annotation file were supplied to the package vcf2gwas (Vogt et al. 2022) which produced Manhattan plots and positional data for putatively causative SNPs.

A region of interest on Cf4 identified via vcf2gwas was then closely examined with respect to gene content and local synteny and collinearity with the *C. quinoa* V2 assembly (Rey et al. 2023). For this purpose, this ∼7-Mb region of interest was manually excised from the full annotation file associated with our ChenoFicP_1.0 assembly, Revigo (Supek et al. 2011) was used to analyze pathways and relationships of genes with attached GO terms, and gene IDs were used to subset the proteome fasta file. BlastP and MCScanX were used to compare this protein subset to the complete proteome of *C. quinoa* and create a homology file. This file, plus the required concatenated BED files, was passed to MCScanX_h, which used default filtering criteria with the exception of MATCH_SIZE, which was set to 9 to only include matches with 10 or more collinear genes. The MCScanX pipeline developed by BDX consulting (Wang et al. 2024) was used to convert data to a format compatible with Circos (Krzywinski et al. 2009).

## Results

### Nuclear genome assembly

The PacBio HiFi read set from *C. ficifolium* “P” comprised a total of 26.5 Gb of sequence with an N50 of ∼14 kb. These data were supplied to Hifiasm version 18, yielding 781 contigs of cumulative length 759,656,873 bp (∼760 Mb) with an average read depth of 34.8 × (Table 1).

**Table 1.** Hifiasm assembly statistics.

Statistics of contigs		Read to contig alignment	
Number of contigs	780	Mapping rate (%)	100.00
Number of contigs > 10,000 bp	780	Split-read rate (%)	0.47
Number of contigs > 1,000,000 bp	9	Depth	34.87
Total length	759,656,810	Mapping rate in large contigs (%)	92.05
Total length of contigs > 1,000,000 bp	729,997,343	Split-read rate in large contigs (%)	0.46
Longest contig	92,986,964	Depth in large contigs	33.45
Second longest contig length	85,613,771	Error and quality metrics	
N50	79,863,811	Small-scale assembly error/per Mbp	0.30
L50	5	QV	62.60

**Fig. 1.**
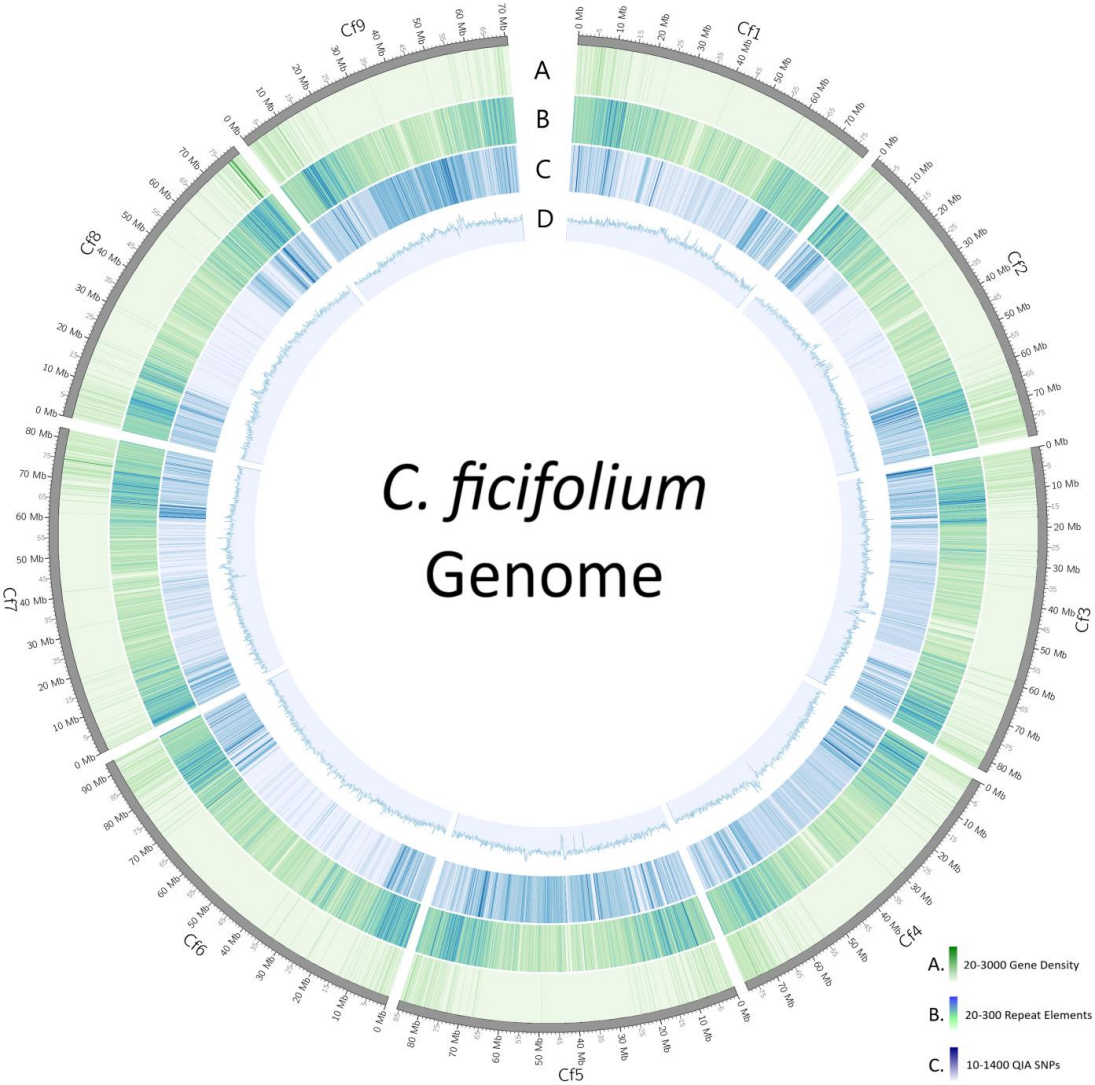
This assembly of the *C. ficifolium* “UNH_ChenoFicP_1.0” nuclear genome comprises 9 PCs and spans 729.9 Mb. (A) Gene density ranging from 20 to 3,000 genes represented per 100-kb window. (B) Repetitive element density ranging from 20 to 300 elements per 100-kb window. (C) SNPs present compared to *C. ficifolium* accession “QC” per 100-kb window. (D) G/C content. Graph represents 20% to 55% G/C.

Of these contigs, the largest 9 ranged in size from a maximum of 92,986,964 bp down to 70,697,261 bp, while the 10th largest (contig ptg000009l) was 8,313,269 bp (Table 2). The remaining 771 contigs were all much smaller: <1 Mbp. On the basis of these size discontinuities, and the prior expectation based on the *C. ficifolium* chromosome number (n = x = 9) (Walsh et al. 2015), the 9 largest Hifiasm contigs were defined as comprising the initial PC assembly. These 9 PCs were then numbered and oriented to maximize correspondence with the *B. vulgaris* EL10.1 reference genome (McGrath et al. 2023), as was done previously in the assembly of the quinoa version 1 assembly (Jarvis et al. 2017). Inspector subsequently identified and corrected 3 structural errors and 228 small-scale assembly errors, reporting a QV score of 63.39 after correction for this assembly (Fig. 1). The G/C content of this genome is 36.9% and varies between ∼20% and 80% per 100-kb window (Fig. 1, ring D). G/C-rich regions are often centrally located within PCs; however, centromeric regions on the whole have an average G/C content, with PC Cf1 having the highest content with 45.47% and the average across all centromeres being 37.75%. When comparing the *C. ficifolium* “QC” accession to the *C. ficifolium* “P” assembly (Fig. 1, ring C), the number of SNPs differentiating the 2 per 100-kb window ranges from as low as 12 to a maximum of 2,321, or ∼1 SNP per 43 bases, as found on PC Cf1 from base 11,800,000 to base 11,900,000.

**Table 2.** Original contig names, PC designations, and contig lengths of the primary assembly plus the 2 largest unplaced contigs after ptg000009l were appended to Cf7.

Name	Renamed	Initial length	Final length
ptg000002l	Cf6	9,298,6964	9,298,6964
ptg000006l	Cf5	85,613,789	85,613,789
ptg000005l	Cf3	82,834,534	82,834,534
ptg000008l	Cf7	74,073,315	82,386,584
ptg000003l	Cf2	79,863,818	79,863,818
ptg000001l	Cf8	79,853,931	79,853,931
ptg000011l	Cf1	77,970,451	77,970,451
ptg000004l	Cf4	77,790,002	77,790,002
ptg000007l	Cf9	70,697,261	70,697,261
ptg000009l	(merged with Cf7)	8,313,269	—
ptg000020l		871,519	871,519
ptg000025l		721,674	721,674

**Fig. 2.**
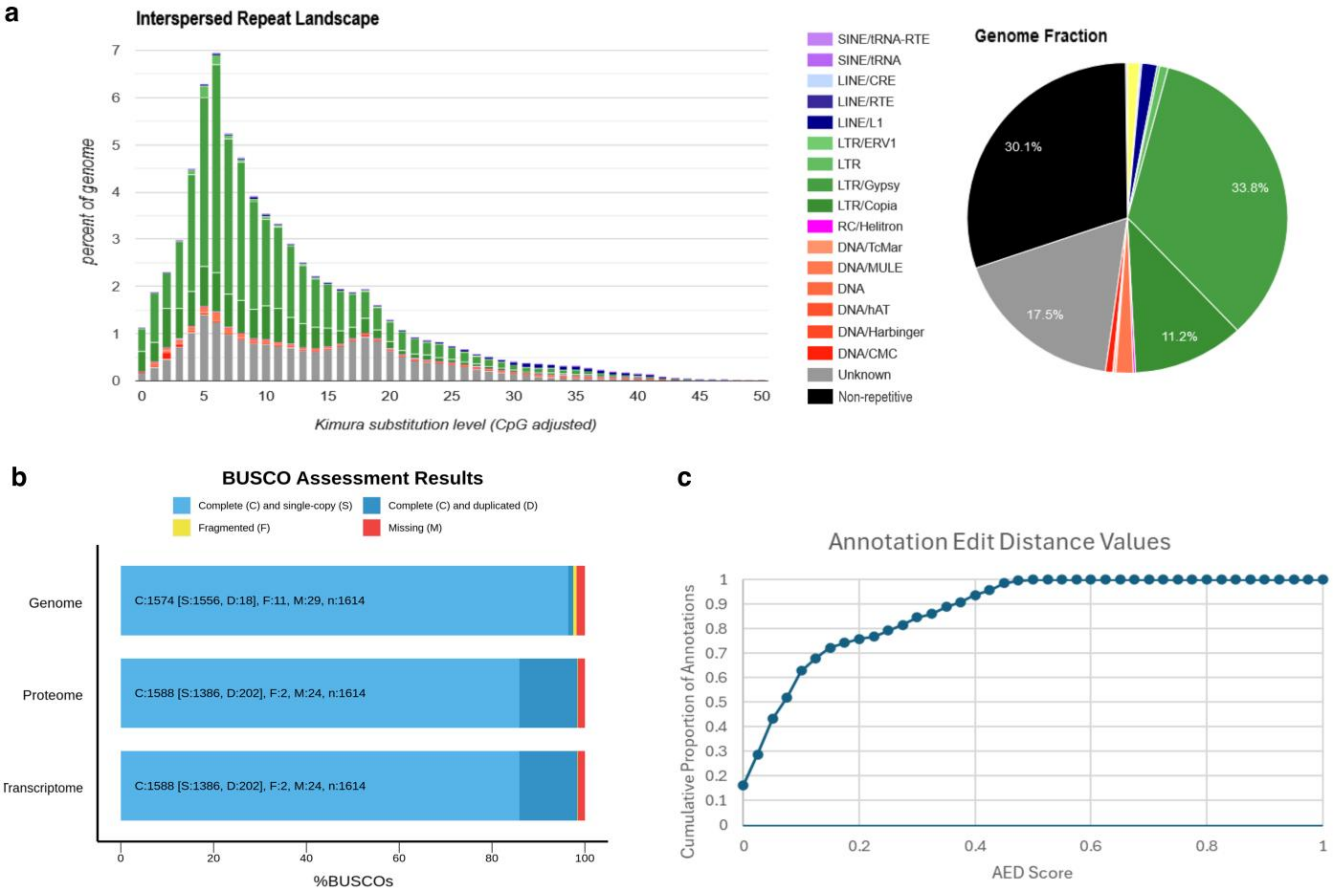
a) Repetitive element analysis and summary graph produced by RepeatModeler. Green (33.8% and 11.2%), LTR elements; gray (17.5%), unknown repetitive elements; black (30.1%), nonrepetitive sequence. b) BUSCO analyses of completeness for the masked genome used in the annotation process (top), the proteome (middle), and transcriptome (bottom). c) AED scores of gene reannotations produced by MAKER. Lower AED values correspond with higher levels of agreement between the annotation and evidence, with 0 representing perfect agreement between the 2.

The initial Hifiasm assembly was telomere-to-telomere for PCs Cf3, Cf4, Cf5, Cf6, Cf8, and Cf9, with Cf1 and Cf2 having only 1 telomere each and Cf7 having a partially assembled telomere which was oriented in a backward direction. A homology search of contig ptg000009l, the only unplaced contig larger than 1 Mbp, relative to the quinoa B subgenome (Rey et al. 2023) revealed that this contig was syntenic to the distal end of quinoa chromosome Cq7B, including a telomeric sequence, and thus directed its placement onto Cf7. Following this adjustment, the final “ChenoFicP_1.0” assembly of 9 PCs had a total length of 729,997,451 bp (∼730 Mb). The CentroMiner package identified 1 region per chromosome as being centromeric (Supplementary Table 2) and produced positional data. Centromeric and telomeric positional data are provided in Supplementary Table 2. BUSCO completeness of the reference genome without unplaced contigs is 96.7% and with unplaced contigs is 97.2% (Fig. 2b).

The remaining 771 unplaced contigs comprised a total of about 29.6 Mb, of which 594 contigs (19.6 Mb) and 6 contigs (626 kb) were subsequently assigned on the basis of sequence homology to the chloroplast and mitochondrial genomes, respectively, leaving a final total of 171 contigs (9.4 Mb) unplaced. Further investigation into unplaced contigs using tidk indicated that both contigs ptg000020 (871,519 bp) and ptg000025 (721,674 bp) contain telomeric sequence. These contigs were found by BWA to likely belong on the upstream ends of Cf2 and Cf7, respectively; however, they have not been included in the ChenoFicP_1.0 assembly pending further confirmation based upon anticipated long-read data.

### Repetitive element identification and genome annotation

Data produced by RepeatModeler indicate that 69.9% of the ChenoFicP_1.0 genome is comprised of repetitive elements, with the largest contributors being LTR/Gypsy (33.8%) and LTR/Copia (11.2%) type repetitive sequences, while unknown repeats account for 17.5% of the total genome fraction. These repetitive elements tend to cluster toward telomeres (Fig. 1, ring B). Nonrepetitive sequence accounts for 30.1% of the genome (Fig. 2a).

The final annotation file was used to calculate relative gene density (Fig. 1, ring A). The BUSCO analysis yielded a transcriptome and proteome completeness of 98.4% (Fig. 2b). Resultant AED values for the final annotation are graphed in Fig. 2c. In total, 22,617 genes were identified and annotated with the average gene length (including introns and UTRs, as defined by MAKER utilizing unprocessed mRNA transcripts) being 3,958 bp.

### Synteny with quinoa B subgenome chromosomes

**Fig. 3.**
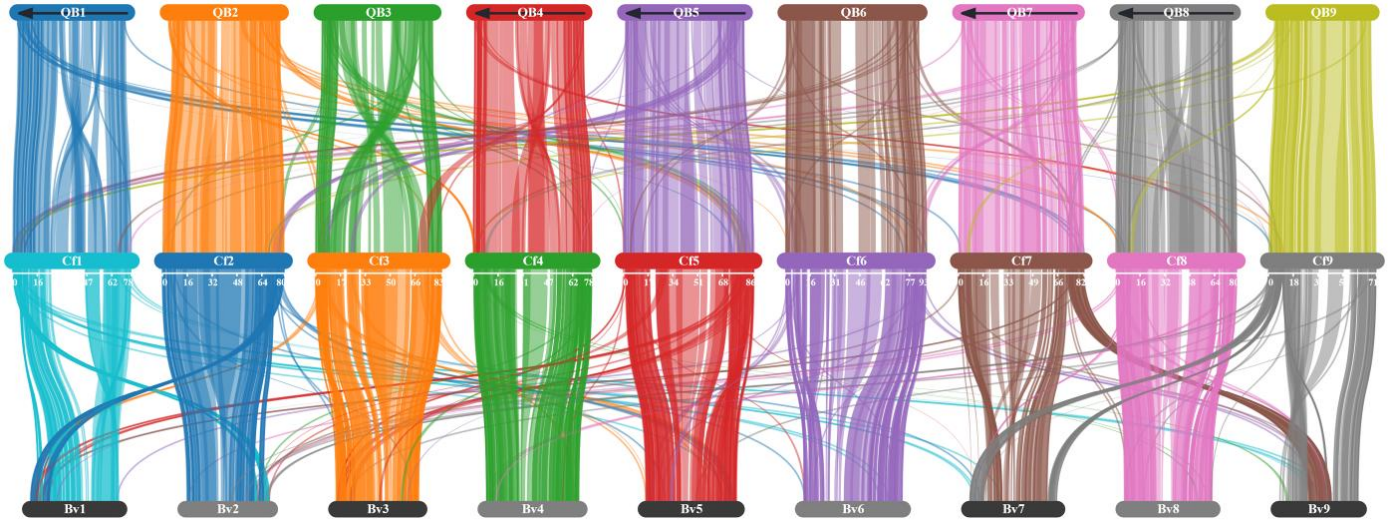
Synteny conservation diagram comparing the assembled PCs of *C. ficifolium* (center) to the B subgenome of quinoa (top) and *B. vulgaris* EL10.1 (bottom). Chromosomes 1, 4, 5, 7, and 8 of the quinoa B subgenome were manually reoriented (flipped—indicated by arrows) to match those of *C. ficifolium* for illustrative purposes.

The MCScanX synteny analysis of the ChenoFicP_1.0 assembly relative to *B. vulgaris* EL10.1 (McGrath et al. 2023) and the quinoa V2 assembly B subgenome (Rey et al. 2023) revealed numerous structural variations. In both comparisons, translocational differences tended to involve small distal regions near telomeres. Large inversional differences were revealed in ChenoFicP_1.0 chromosomes Cf1, Cf3, and Cf4 relative to the quinoa B subgenome homologs, the largest of which was on Cf3 and spanned ∼63.2 Mb (Fig. 3). These inversions were positioned centrally and in every case for chromosomes Cf1, Cf3, and Cf4 were pericentric.

### Chloroplast genome assembly

**Fig. 4.**
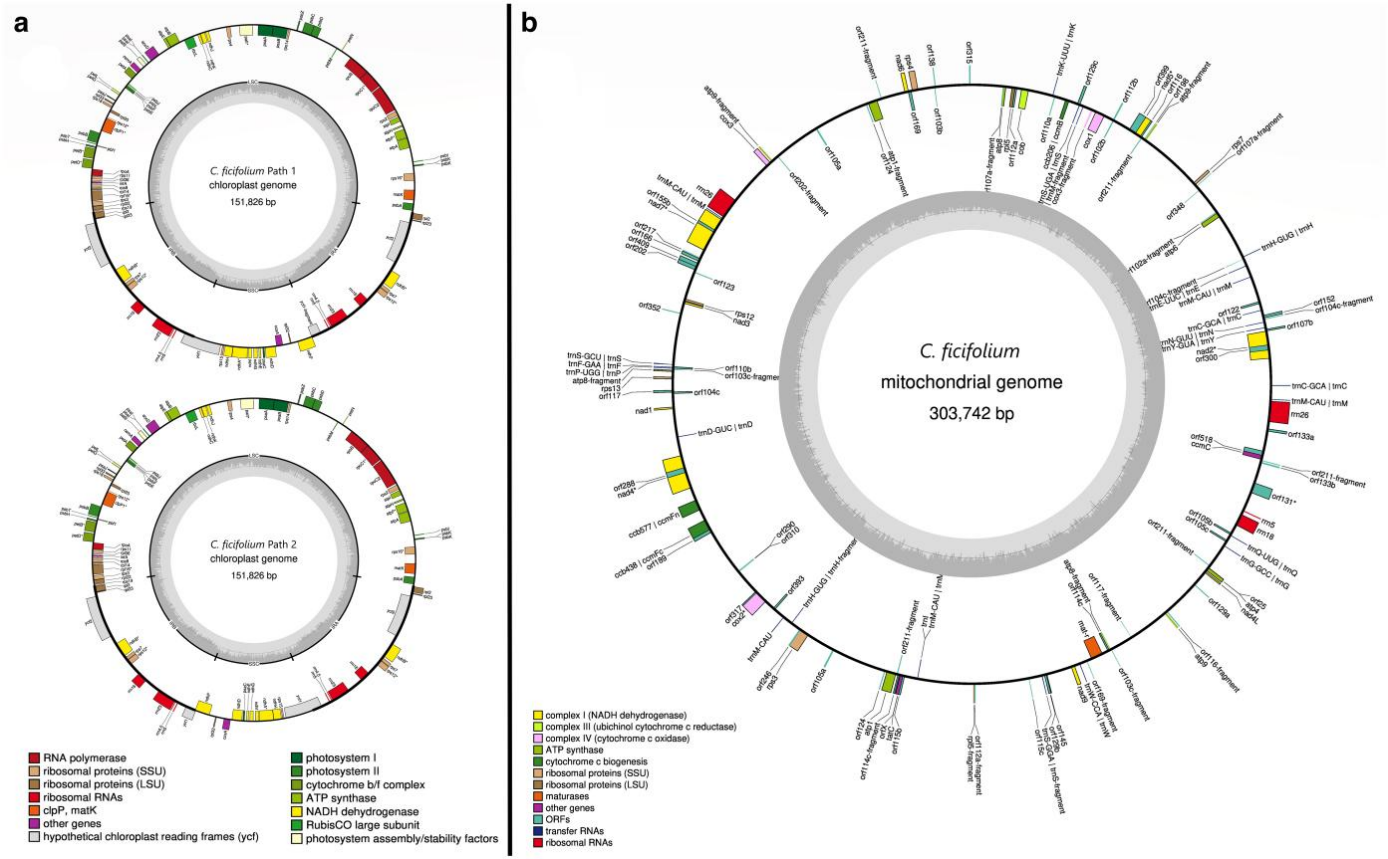
a) Both assembly versions of the *C. ficifolium* chloroplast genome represented by “path 1” and “path 2.” These assemblies differ only in the direction of the SSC region. “Path 2” (lower) was concordant with the *C. quinoa* chloroplast genome (MK159176). Genes inside the circle are transcribed in the clockwise direction; genes outside are transcribed counterclockwise. b) Gene map of the *C. ficifolium* mitogenome. The mitochondrial genome contains 118 protein-coding genes, 23 tRNA genes, and is ∼304 kb in length.

GetOrganelle produced 2 versions of the *C. ficifolium* chloroplast genome assembly, termed “path 1” and “path 2,” both 151,826 bp in length, but differing in their orientations of the short single copy (SSC) region relative to the long single copy (LSC) region (Fig. 4a). When supplied with ∼263 Mb of Nanopore reads, Cp-hap (Wang et al. 2019) returned approximately equal long-read support for both path 1 (248 reads) and path 2 (242 reads) orientations, where the path 2 orientation (Fig. 4a, lower) coincided with that of the quinoa chloroplast assembly (MK159176.1) as reported by Maughan et al. (2019).

In both path 1 and path 2 orientations, the LSC region is 83,900 bp, the SSC is 18,168 bp, and each of the inverted repeat regions is 15,109 bases in length. The chloroplast genome regional boundaries are specified in Table 3. The assembly, including both inverted repeat regions, has a GC content of 37.3% and comprises 131 genes, of which there are 86 protein-encoding, 37 tRNA, and 8 rRNA genes. For comparison, the previously published (Kim, Chung, et al. 2019; Kim, Park, et al. 2019) *C. ficifolium* chloroplast genome assembly (MK182725) had 129 genes, including 84 protein-encoding, 37 tRNA, and 8 rRNA genes.

**Table 3.** Start and end positions of the 4 regions of the chloroplast assembly.

Cp region	Start	End	No. of genes	Protein encoding
LSC	1	83,670	85	63
IR	83,671	108,779	16	5
SSC	108,780	126,717	14	13
IR	126,718	151,826	16	5
		Total	131	86

Note that these positions apply to both path 1 and path 2 haplotypes; however, the SSC region is inverted in path 1 relative to path 2.

### Mitochondrial genome assembly

A 275-kb contig containing a partially assembled mitochondrial genome from the “P” accession was initially identified from a preliminary Hifiasm assembly. In an iterative sequence seeding process, Nanopore reads were aligned to this contig, extracted from the resulting alignment, and assembled de novo, and reads were once again aligned. After 2 rounds of this process, a contig was produced that was manually circularized and polished with NextPolish using HiFi reads. The resulting “P” mitochondrial genome assembly has a GC content of 43.8% and comprises 90 genes, of which 33 are protein-coding genes (Fig. 4b). The *C. ficifolium* mitochondrial genome assembly is similar in length to that of *C. quinoa* (MK182703), at 303,742 bp vs 319,505 bp, respectively, and in protein-coding genes, with 33 and 30, respectively (Maughan et al. 2019), although these 2 assemblies display several structural differences.

### Organelle genomes’ modes of transmission

**Fig. 5.**
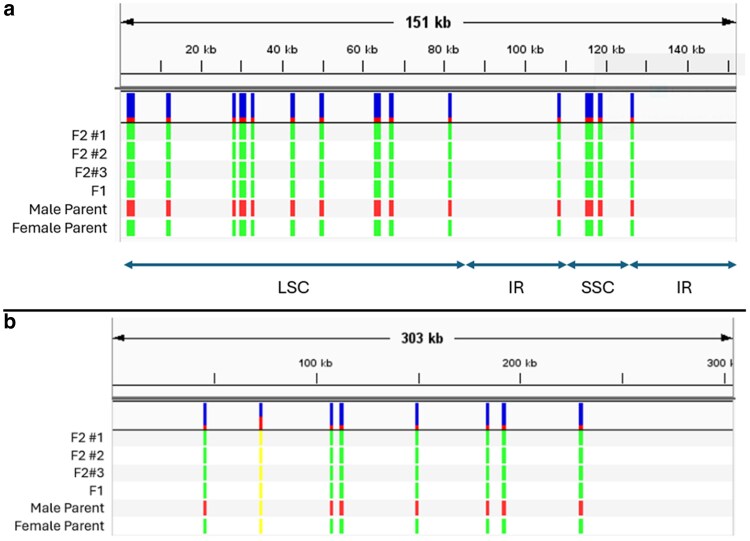
a) Chloroplastic “path 2” polymorphisms found among 3 F2 individuals, 1 F1 individual, and the maternal (“QC”) and paternal (“P”) parents in the cross that yielded the F1 individual. b) Mitochondrial polymorphisms found among 3 F2 individuals, 1 F1 individual, and the male and female within the cross that yielded the F1 individual, “QC” and “P,” respectively. Interestingly, 1 polymorphism shared across all individuals is a heterozygous A/C SNP.

When plastid genome mode of transmission was examined using multisample vcf files containing both parents, “P” and “QC,” an F1, and 4 F2 individuals, 2 distinct sequence haplotypes were revealed. Of the respective polymorphisms, 2 were single nucleotide indels, while the remaining 17 were SNPs (Supplementary Table 3). The F1 and all F2 individuals share the same sequence haplotype as the maternal parent (“P”) as distinct from the “QC” paternal haplotype (Fig. 5a), thereby demonstrating maternal transmission of the chloroplast genome in the “P” × “QC” cross used to generate the respective F1 and F2 progenies.

To determine the transmission pattern of the mitochondrial genome in the “P” × “QC” cross, “QC” short-read alignment to the “P” mitochondrial reference genome detected 9 polymorphisms, of which 8 were informative, with the uninformative polymorphism being a SNP for which all individuals had a nonreference nucleotide. Seven of those informative polymorphisms were SNPs, while the eighth was a 22-bp indel (Fig. 5b; Supplementary Table 3). These data demonstrate that transmission of the mitochondrial genome in the “P” × “QC” cross was maternal.

### Chloroplast and mitochondrial genome ancestries

**Fig. 6.**
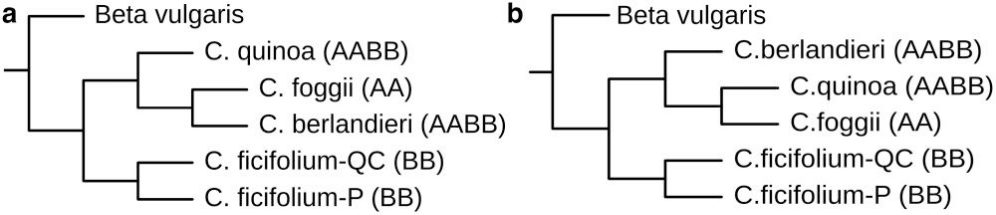
a) Phylogenetic tree illustrating chloroplast genome ancestral relationships among *Chenopodium* AA and BB diploids and AABB tetraploids in our study. This phylogenetic tree is based on chloroplast assemblies from *C. foggii*, both *C. ficifolium* accessions, *C. quinoa*, *C. berlandieri*, and *B. vulgaris* which is set as the outgroup. b) Phylogenetic tree illustrating mitochondrial genome ancestral relationships among *Chenopodium* AA and BB diploids and AABB tetraploids. This phylogenetic tree was constructed using reference-guided assemblies with the *C. quinoa* mitogenome (NC_041093.1) selected as the reference genome. *B. vulgaris* is set as the outgroup.

A phylogeny based on de novo assembled chloroplast genomes from *B. vulgaris*, *C. quinoa*, *C. berlandieri* var. *macrocalycium*, *C. foggii*, and both the “P” and “QC” *C. ficifolium* accessions (Fig. 6a) revealed a close affinity of the 2 *Chenopodium* allotetraploids with the A genome diploid *C. foggii* and a more distant relationship with B genome diploid *C. ficifolium*. Similarly, mitochondrial reference-based assemblies utilizing the same set of germplasm (Fig. 6b) in a narrowly focused phylogeny detected the same pattern of affinities. These outcomes support the prior hypothesis (Maughan et al. 2019) that the organelle genomes of the AABB allotetraploids were both contributed by the AA ancestral diploid.

### GWAS

**Fig. 7.**
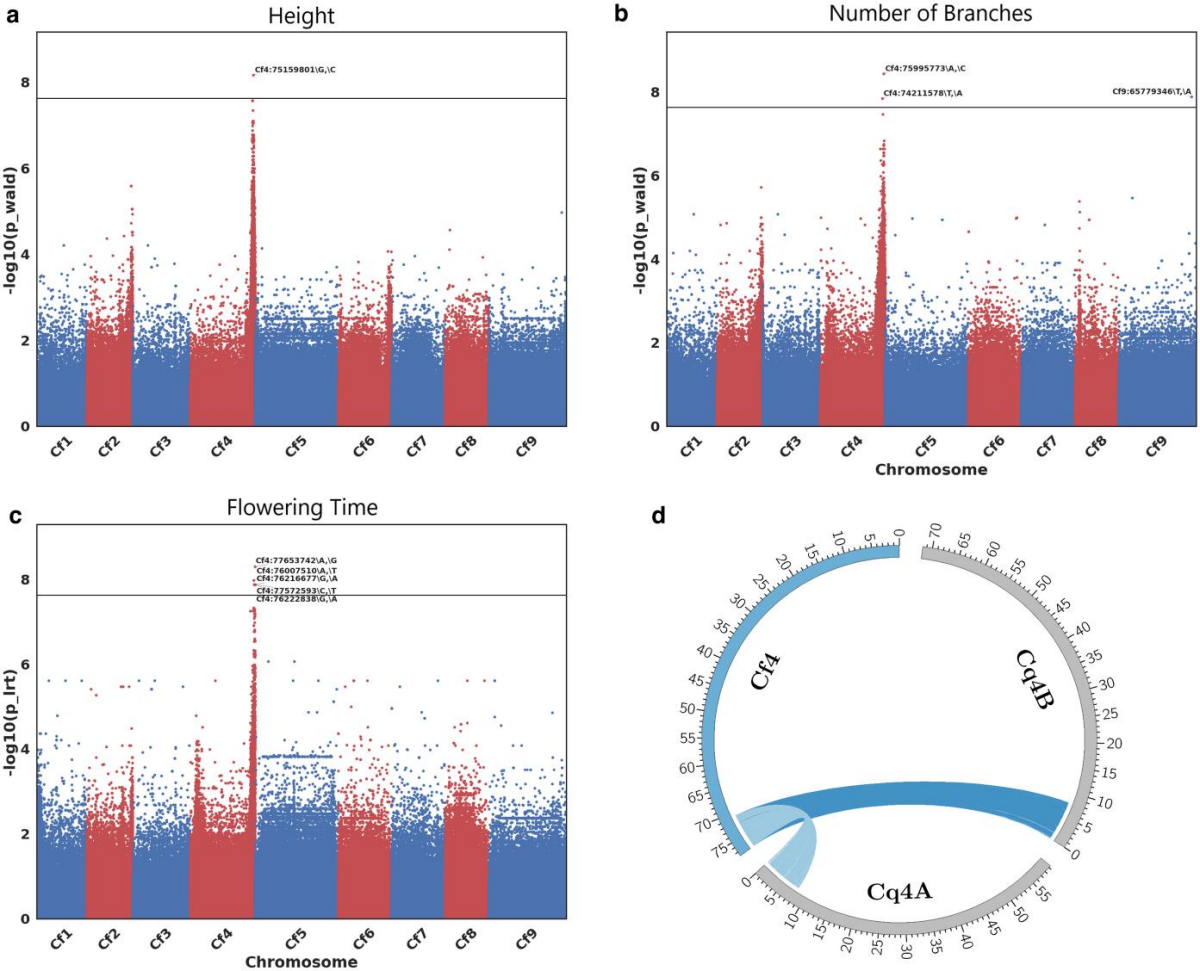
a to c) GWAS Manhattan plots indicating regions of the genome implicated in the control of plant height, number of branches, and flowering time. d) Synteny analysis of the ∼7-Mb region found in *C. ficifolium* Cf4 (left) implicated by the GWAS as compared to *C. quinoa* chromosomes Cq4A and Cq4B as oriented in Rey et al. (2023). Both quinoa chromosomes Cq4A and Cq4B are in reverse orientation compared to *C. ficifolium* chromosome Cf4.

Of the 5 trait-specific GWAS analyses performed, 2 (branch angle and internode length) failed to detect genomic associations. The remaining 3 traits—days to flowering, plant height, and branch number—were found by GWAS to correlate with a single common region of chromosome Cf4 (Fig. 7a to c). Of these 3 traits, the correlated SNPs for branch number covered the largest region and fully contained those implicated in the plant height and days to flower analyses. The implicated region spans 7,020,561 bp between positions 70,765,247 and 77,785,808 on Cf4. Features within this region were pulled from the annotation file to create a list of gene names and GO terms associated with those genes. There are 770 annotated genes within the defined region. Of those, 132 were of unknown function. GO terms were assigned to 403 genes, including *FTL1*, which at position 74,807,401 to 74,811,571 is number 425 on the list of 770 genes and is annotated as *Vernalization 3* (*VRN3*). *FTL1* had 1 product classification identified by Interpro (Paysan-Lafosse et al. 2023), being a phosphatidylethanolamine-binding protein (IPR008914).

A microsynteny analysis focusing on the ∼7-Mb window implicated in this GWAS was performed to identify homeologs within both *C. quinoa* subgenomes and their locations (Fig. 7d). MCScanX identified 9 collinear blocks of 10 or more genes from this region in *C. ficifolium* that also occur, but in reverse orientation, on quinoa chromosomes Cq4A and Cq4B of the Rey et al. (2023) assembly, as will be discussed later. The analysis of these data indicates that for the 770 genes found in *C. ficifolium* annotation file at this ∼7-Mb region, there were 581 homoeologous genes found within the *C. quinoa* A subgenome and 610 found within the B subgenome. In total, 107 of the *C. ficifolium* genes within this region are of unknown function, and of those, only 6 have associated GO terms.

## Discussion

### Nuclear genome

As described here, the ChenoFicP_1.0 assembly provides a valuable component to the *C. ficifolium* diploid (BB) model system, as will be expanded upon in subsequent reports on ongoing investigations at the diploid and allotetraploid (AABB) levels in *Chenopodium*. PCs within this *C. ficifolium* assembly have been numbered to correspond with, and oriented to be syntenic and collinear with, the previously published *B. vulgaris* (2*n* = 2*x* = 18) EL10.1 genome (McGrath et al. 2023), thereby following the example of Jarvis et al. (2017) in which the quinoa V1 assembly was similarly oriented. The fact that the quinoa V2 assembly (Rey et al. 2023) did not follow this orientation pattern opens the possibility for some confusion, especially with respect to chromosomes Cq1B, Cq4B, Cq5B, Cq7B, and Cq8B, which are presented and numbered in reverse orientation in quinoa V2 as compared with quinoa V1 and beet EL10.1. We have followed the initial orientation approach in conjunction with the recently published *Chenopodium* pangenome analysis (Jaggi et al. 2024), which employs the original orientation pattern.

Beet was initially selected as the genome comparator for *Chenopodium* because it was the first species to have a published reference genome within the subfamily Chenopodioideae. While the diploid *Spinacia oleracea* (2*n* = 2*x* = 12) now has a published genome (Cai et al. 2021), its nuclear genome has only 6 chromosomes, making comparisons with it suboptimal for identifying syntenic relationships and orienting chromosomes of newly sequenced species. Genome assemblies have been reported for 3 other *Chenopodium* diploids: the AA diploids *Chenopodium pallidicaule* (Jarvis et al. 2017) and *Chenopodium watsonii* (Young et al. 2023) and the BB diploid *C. suecicum* versions 1 (Jarvis et al. 2017) and 2 (Rey et al. 2023). However, to our knowledge, no gene–trait association studies have yet been conducted in these species.

The ChenoFicP_1.0 9-PC assembly size of ∼730-Mbp incorporates 96% of the 760-Mbp total assembly for the 781 contigs generated by Hifiasm. Cytometrically determined C-values of *C. ficifolium* “P” predict a genome size in the range of 821 to 831 Mbp (Neff 2017). For comparison, the 9 B subgenome PCs of the quinoa assembly sum to 670 Mbp in length (Rey et al. 2023), or ∼8% smaller than the ChenoFicP_1.0 assembly. Similarly, the 9 PCs of the quinoa A subgenome sum to 531 Mbp (Rey et al. 2023), which is 3% smaller than the diploid *C. watsonii* 548-Mbp A genome assembly (Young et al. 2023). An earlier, broad survey of *Chenopodium* germplasm reported C-values of 893 Mbp for a Eurasian accession of *C. ficifolium*, 645 Mbp for *C. watsonii*, and 1.545 Gbp for *C. quinoa* (Mandák et al. 2016) Thus, all 3 of the aforementioned *Chenopodium* genome assemblies are somewhat smaller than the size predicted by cytometric assay. It has been speculated that this pattern of discrepancy may be due to incomplete assembly coverage of some repetitive regions (Young et al. 2023), or it could be due to a consistent technical artifact of flow cytometric analyses.

The ChenoFicP_1.0 genome contains 22,617 genes (total genes), of which 5,060 encode proteins that have yet to be characterized within the UniProtKB/Swiss-Prot database. Gene density is greatest in the distal regions of chromosomes (Fig. 1, ring A). For comparison, the total gene counts of the *C. quinoa* A and B subgenomes are 25,913 and 26,942, respectively (Rey et al. 2023), the AA diploid *C*. *watsonii* count was 30,325 (Young et al. 2023), and the BB diploid *C. suecicum* count was 29,702 (Rey et al. 2023). Compared with the ChenoFicP_1.0 assembly, the total number of annotated genes within the *C. quinoa* B subgenome is ∼19.1% higher. Given that different gene count methodologies were used in these various studies, the observed differences in gene counts among subgenomes and species should be interpreted with caution.

RepeatModeler indicated that repetitive elements account for ∼70% (510 Mbp) of the *C. ficifolium* genome. For comparison, the quinoa repetitive element content was ∼65% for the total genome (Zou et al. 2017; Rey et al. 2023) and ∼63% (421 Mbp) for the B subgenome, with the largest contributors being long terminal repeat retrotransposons representing 46% of the total genome size. Over half of the *C. ficifolium* repetitive elements are retrotransposons that cluster toward the center of chromosomes (Fig. 1, ring B). These data indicate that there has been some change in the repetitive element landscape in *C. ficifolium* and/or the *C. quinoa* B subgenome subsequent to the quinoa allotetraploid speciation event.

### Organelle genome assemblies

The generation of 2 different chloroplast genome assembly versions, designated paths 1 and 2, was an unexpected outcome. Prior chloroplast genome assemblies in *Chenopodium*, including *C. quinoa* (Maughan et al. 2019; Gao et al. 2021) and *C. ficifolium* (Kim, Chung, et al. 2019), and in the broader Amaranthaceae family (Chaney et al. 2016; Dong et al. 2016; Kim, Chung, et al. 2019; Ding et al. 2021) including sugar beet (McGrath et al. 2023), have reported only a single chloroplast genome configuration, corresponding to our path 2 assembly, although Kim, Park, et al. (2019) mention the need to reposition the SSC and LSC regions of the quinoa accession “Real Blanca” for their alignment procedure, indicating a disagreement in SSC and/or LSC orientation when compared to other assemblies. However, recent literature (Wang et al. 2019) (and citations therein) has established that chloroplast genomes do indeed exist in 2 equally represented alternate configurations in many if not most plant species, as has now become evident in *Chenopodium* and that the detection of these alternate forms is enhanced by employment of long-read sequencing technologies such as PacBio HiFi and Nanopore. The very long and accurate reads now available from Nanopore technology will be of particular value in relation to mitochondrial genomes, the potential alternate forms of which are often challenging to assemble (Kozik et al. 2019). Given the known complexities of mitochondrial genome organization, the assembly configuration that we present here should be viewed as preliminary and subject to revision.

### Organelle transmission patterns

In the absence of evidence to the contrary, organelle genomes are generally assumed to be transmitted maternally in angiosperms; however, exceptions have been reported (Davis et al. 2010), including paternal chloroplast transmission in some *Actinidia* hybrids (Testolin and Cipriani 1997). Until very recently (Maughan et al. 2024), empirical documentation of modes of organelle inheritance in *Chenopodium* appears to have been limited to a single study involving atrazine resistance in *Chenopodium album* (Warwick and Black 1980; Corriveau and Coleman 1988; Harris and Ingram 1991; Kolano et al. 2016). Our analysis agrees with this prior study in its finding of maternal transmission of both chloroplast and mitochondrial markers in our diploid level *C. ficifolium* cross. Similarly, Maughan et al. (2024) have documented the maternal transmission of the organelle genomes in several tetraploid level *Chenopodium* crosses.

### Organelle genome ancestries

Although *C. ficifolium* is a candidate B nuclear genome donor in the initial hybridization that formed quinoa 3.3 to 6.3 million years ago (Štorchová et al. 2015; Kolano et al. 2016; Jarvis et al. 2017; Maughan et al. 2019), prior analyses indicate that a BB diploid was not the ancestral donor of the allotetraploids’ organelle genomes (Maughan et al. 2019). Instead, that role has been assigned to the A nuclear genome donor (Maughan et al. 2019). That assignment is supported by our chloroplast and mitochondrial minimal phylogenies, which both place *C. ficifolium* as sister to a clade consisting of AABB tetraploids and an AA diploid.

It is considered likely that, in the initial AABB hybridization event, the ancestral AA diploid served as the female and maternal organelle genome donor (Maughan et al. 2019). Documentation of such in our *C. ficifolium* cross and in tetraploid level crosses (Maughan et al. 2024) strengthens the thesis that the original hybridization event was of the form AA × BB, i.e., with the AA diploid ancestor serving as the female. Final validation of this model will await the analysis of organelle genome transmission in reciprocal crosses between AA and BB diploids.

### GWAS

The data produced from these genome-wide analyses indicate that, in the studied segregating population, variation in the agronomically important traits of days to flower, plant height, and branch number was predominantly, if not entirely, under the control of 1 or more genes within a single, 7-Mb region located toward the bottom of PC Cf4 and containing the *FTL1* locus previously employed as a marker by Subedi et al. (2021). Our genome-wide analysis extends the findings of Subedi et al. (2021) by focusing attention on this Cf4 “hot spot” and the genes residing therein. Of note, the association of branch angle with a single SNP on Cf9 was not further investigated due to its presumed spurious nature. Manhattan plots will typically associate a number of SNPs within and surrounding a causative region, and this questionable SNP did not have any significantly associated markers adjacent to it, nor was it seen in the other 2 analyses investigating plant height or days to flower.

GO data submitted to Revigo indicated the presence of a myriad of genes responsible for controlling functions that are not intrinsically associated with these traits of interest; however, this region contains *FTL1*, which was automatically annotated as *VRN3* within the GFF file. This gene is between positions 74,807,401 and 74,811,571, placing it centrally within the denoted significance region on Cf4. *FTL1* had 1 product classification identified by Interpro (Paysan-Lafosse et al. 2023), being a phosphatidylethanolamine-binding protein (IPR008914).

To further understand relationships between *C. ficifolium* and *C. quinoa* and functionally utilize *C. ficifolium* as a model species, BlastP and MCScanX microsynteny analyses were performed to investigate relative gene locations across species and subgenomes (Fig. 7d). When comparing the *C. ficifolium* region of interest to both of the quinoa subgenomes, all homologous genes in syntenic blocks of 10 or more genes were found to reside in corresponding distal regions of quinoa chromosomes Cq4A and Cq4B, reflecting substantial gene order and location conservation within the respective genomic window. This analysis identified quinoa genes CQ035263 (A subgenome) and CQ019992 (B subgenome) within the *C. quinoa* V2 annotation (GFF3) file as being homologous to *C. ficifolium VRN3* (*FTL1*). The product of CQ035263 was queried with BlastP and has an 83% sequence identity match to XP_021772286.1, *C. quinoa FLOWERING LOCUS T-like*. Similarly, the product of gene CQ019992 is identical to XP_021734330.1. *C. quinoa FLOWERING LOCUS T-like* has a 97% protein identity with the annotated *C. ficifolium* gene Cfic_20093. The data produced using *C. ficifolium* as a model organism for *C. quinoa* via this pipeline have been demonstrated to be an efficacious way of establishing gene–trait relationships via GWAS and locating syntenic regions across species that may be worth investigating to understand agronomically relevant traits. Further investigation of genes within this region is required, perhaps in conjunction with more high-depth sequence data and additional plants to facilitate a fine-mapping study; however, these data are strongly indicative of 1 or more genes within this region as being implicated in the control of these traits. Further research, likely employing gene editing or transformation methods, is required to test this hypothesis and firmly establish this relationship.

The results of this study, in particular the synteny and collinearity analysis comparing *C. ficifolium* and quinoa “QQ74” chromosomes, present intriguing hints regarding the potential usefulness of figleaf goosefoot as a breeding resource for quinoa improvement. The primary structural variants differentiating the B genome chromosomes in these 2 species are 3 large pericentric inversions. However, the largest of these, on chromosome Cq3B, was a rearrangement present only in a subset of quinoa lines derived from lowland Chilean germplasm, with at least 80% of quinoa lines expected to have collinear Cq3B in comparison with figleaf goosefoot (Rey et al. 2023). The remaining 2 inversions, on Cq1B and Cq4B, appear to have breakpoints extending less than halfway out into their respective chromosome arms, such that they do not encompass recombinogenic regions (Maughan et al. 2024). Consequently, while triploid (AB_q_B_f_) figleaf goosefoot × quinoa hybrids are expected to be highly sterile, backcross hybrids might theoretically be recoverable and, if so, we might expect a high degree of pairing and recombination between their B genomes. This approach—crossing of the polyploid crop to its wild, diploid progenitor—has been successfully employed to introduce numerous resistance genes from the wild progenitor goatgrass (*Aegilops tauschii*, DD) into bread wheat (*Triticum aestivum*, AABBDD) while restricting recombination, and consequently undesirable linkage drag, to a single subgenome (Cox et al. 1994).

## Conclusion

The development of high-quality annotated reference nuclear and chloroplast genomes and a preliminary mitochondrial genome for *C. ficifolium* serves as a foundational advance in the development of this species as a BB diploid model system for related allotetraploids of interest, with a primary focus on *C. quinoa* and *C. berlandieri.* Looking ahead, next steps in the development and implementation of this model system include broadening the germplasm base via plant collection in diverse environments; establishing new hybrids and populations segregating for additional traits of interest, including male sterility (Ward 1998) and disease resistance, specifically downy mildew (Nolen et al. 2022); and the establishment of effective protocols for gene editing and in vitro culture and regeneration for both quinoa and its diploid model(s), with immediate focus on manipulation of *FTL1*. The fact that the organelle genomes present in allotetraploid quinoa were likely derived via maternal transmission from its AA diploid ancestor, not from its BB ancestor, points to the need for parallel development of an AA diploid model system in *Chenopodium*. Foundational steps in this direction have already been taken via the generation of genome assemblies for AA diploids *C. pallidicaule* (Mangelson et al. 2019) and *C. watsonii* (Young et al. 2023) and useful germplasm collection in *C. foggii* (Neff, Sullivan and Davis 2018). As yet, to our knowledge, no genetic crosses or segregating progeny populations have yet been generated in an AA diploid, so this should be a top priority going forward. Finally, opportunity exists for breeding of quinoa with figleaf goosefoot as an avenue for gene introgression from the diploid to the tetraploid level.

## Supplementary Material

jkaf162_Supplementary_Data

## Data Availability

The reference genome and sequence datasets used to create it are available from NCBI using the BioProject ID PRJNA1101583, assembly accession GCA_046866025.1/WGS project JBDLLL010000000. The reference genome can be found using the search term “UNH_ChenoFicP_1.0.” The reference assembly, annotation data in GFF3 format, and supplementary annotation files can be accessed via figshare.com: https://doi.org/10.6084/m9.figshare.c.7590620. Sequence data for F2 individuals used in GWAS analyses are hosted on NCBI: https://www.ncbi.nlm.nih.gov/bioproject/PRJNA1262536. Code used can be found on GitHub: https://github.com/UNH-DavisLab/Ficifolium-Genome-V1/. Supplemental material available at G3 online.
