# Supplementary material for: The genome of Chenopodium ficifolium: developing genetic resources and a diploid model system for allotetraploid quinoa
Source: G3 (Bethesda). 2025 Jul 18;15(10):jkaf162. doi: 10.1093/g3journal/jkaf162 (PMC12506657; doi:10.1093/g3journal/jkaf162)
Supplement: jkaf162_Supplementary_Data [file jkaf162_supplementary_data.zip › Supplementary_Table_Legends_G3-2025-405921.docx]

**Supplementary Tables**

Supplementary Table 1. Individuals included in GWAS and phenotypic data used in analyses.

Supplementary Table 2. Centromere positions and lengths as identified by quarTeT CentroMiner and telomere lengths and directions as identified by quarTeT TeloExplorer.

Supplementary Table 3. Positions and alleles of SNPs found in mitochondrial and chloroplast genomes used to establish maternal cytoplasmic inheritance.
